# How is Excitotoxicity Being Modelled in iPSC-Derived Neurons?

**DOI:** 10.1007/s12640-024-00721-3

**Published:** 2024-10-15

**Authors:** Jan L. Cheng, Anthony L. Cook, Jana Talbot, Sharn Perry

**Affiliations:** Wicking Dementia Research and Education Centre, College of Health and Medicine, University of Tasmania, 17 Liverpool Street, Hobart, TAS Australia

**Keywords:** DREADDs, Kainic acid, Calcium, Glutamate, NMDAR, AMPAR

## Abstract

Excitotoxicity linked either to environmental causes (pesticide and cyanotoxin exposure), excitatory neurotransmitter imbalance, or to intrinsic neuronal hyperexcitability, is a pathological mechanism central to neurodegeneration in amyotrophic lateral sclerosis (ALS). Investigation of excitotoxic mechanisms using in vitro and in vivo animal models has been central to understanding ALS mechanisms of disease. In particular, advances in induced pluripotent stem cell (iPSC) technologies now provide human cell-based models that are readily amenable to environmental and network-based excitotoxic manipulations. The cell-type specific differentiation of iPSC, combined with approaches to modelling excitotoxicity that include editing of disease-associated gene variants, chemogenetics, and environmental risk-associated exposures make iPSC primed to examine gene-environment interactions and disease-associated excitotoxic mechanisms. Critical to this is knowledge of which neurotransmitter receptor subunits are expressed by iPSC-derived neuronal cultures being studied, how their activity responds to antagonists and agonists of these receptors, and how to interpret data derived from multi-parameter electrophysiological recordings. This review explores how iPSC-based studies have contributed to our understanding of ALS-linked excitotoxicity and highlights novel approaches to inducing excitotoxicity in iPSC-derived neurons to further our understanding of its pathological pathways.

## Introduction

**Fig. 1:**
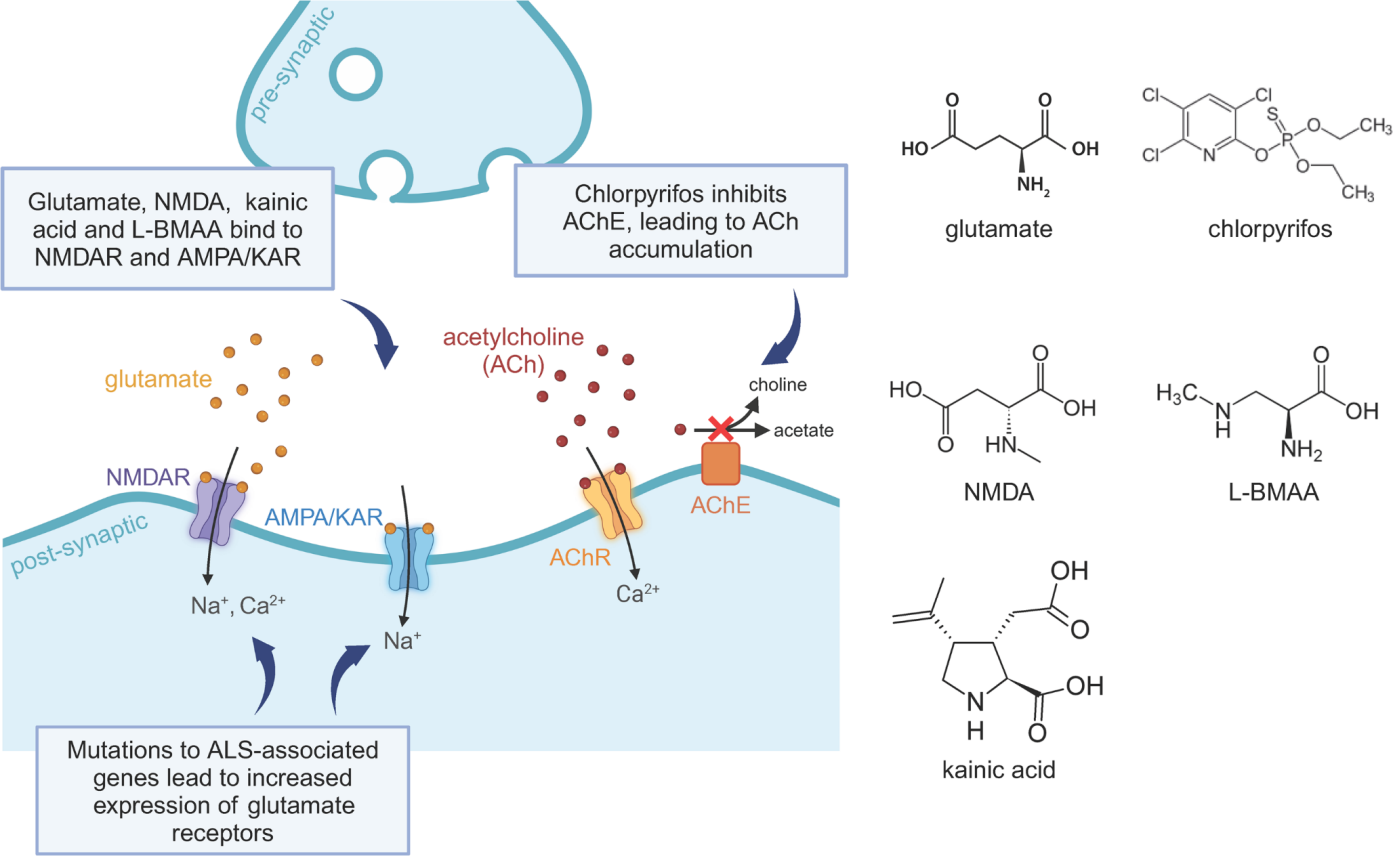
Generating excitotoxicity in ALS disease models. Excitotoxicity can be induced through application of glutamate, NMDA, and other structurally similar glutamate receptor agonists, such as KA and beta-methylamino-L-alanine (L-BMAA), that bind with NMDA and AMPA/KA receptors. Mutations to ALS-associated genes such as SOD1, C9ORF72, TARDBP, and FUS, lead to upregulated expression of glutamate receptors, increasing neuron sensitivity to glutamate-mediated activation. Excitotoxicity can also be induced in neurons through non-glutamate receptor pathways, such as through chloropyrifos-induced, indirect activation of cholinergic receptors

Excitotoxicity is a pathological mechanism common to many neurodegenerative diseases, including amyotrophic lateral sclerosis (ALS). Excitotoxicity causes neuronal degeneration through the toxic actions of excitatory neurotransmitters, primarily glutamate, leading to the excess influx of calcium ions, triggering loss of neuron structure and cell death (Bursch et al. 2019; Dafinca et al. 2020; Gregory et al. 2020; Selvaraj et al. 2018; Van Den Bosch et al. 2006). ALS is associated with many different environmental causes (Al-Chalabi et al. 2014; Al-Chalabi and Lewis 2011; Chiò et al. 2018; Goutman et al. 2023), with excitotoxicity and the hallmark pathology of TDP-43 mislocalisation being common features resulting from exposure to cyanotoxins such as cyanotoxin L-BMAA (Anzilotti et al. 2023; Rao et al. 2006) and pesticides such as chlorpyrifos (Pulkrabkova et al. 2023; Rush et al. 2010) (Fig. 1). Such environmental agents are readily amenable to laboratory investigations using human induced pluripotent stem cell (iPSC)-derived models, and can provide insight into neuronal subtype-specific vulnerability to degeneration, downstream signaling processes that mediate axon degeneration and cell death, and the capacity of neurons to functionally and morphologically recover from excitotoxic insults. Contemporary techniques in functional analysis of neuronal network activity, gene editing, and high-content imaging enable generation of detailed data from iPSC-based models of excitotoxicity that can be mined to refine hypotheses regarding pathological mechanisms and to identify potential therapeutic targets.

Hyperexcitability, an increased propensity of neurons to generate an action potential in response to stimuli, has been extensively described in ALS patients and animal models. It is considered a pathogenic mechanism central to development of ALS, where the excitotoxic effects of excess amino acid neurotransmitters, especially glutamate, lead to the selective death of vulnerable neurons via the overactivation of neurotransmitter receptors allowing influx of calcium ions (King et al. 2016; Odierna et al. 2024). In people with ALS, hyperexcitability occurs throughout the nervous system, with both cortical hyperexcitability (Vucic and Kiernan 2006) and neuromuscular junction (NMJ) hyperexcitability (Noto et al. 2023) having been described. Hyperexcitability is also the target of therapeutic strategies for ALS, with riluzole, one of only three approved small-molecular drugs for ALS, acting to reduce glutamatergic neurotransmission and thereby slow disease, albeit with small clinical effect (Bensimon et al. 1994).

Investigation of hyperexcitability and excitotoxicity in animal models, notably the SOD1^G93A^ transgenic mouse, and models based on TDP-43 variants or forced mislocalisation, has been a focal point in ALS research (e.g. (Pieri et al. 2003; Dyer et al. 2023; Fogarty et al. 2016). These models have generated many hypotheses regarding the origin of hyperexcitability in ALS, including imbalance of neurons and interneurons (Clark et al. 2017), and corticomotor mislocalisation of TDP-43 causing hyperexcitability, leading to spinal motor neuron degeneration (Reale et al. 2023). Experiments using primary cultures from these transgenic or wildtype mice exposed to excitotoxins (e.g. kainic acid (KA) have complemented these studies, providing the basis for our understanding of the subcellular events caused by hyperexcitable or excitotoxic environments, and revealed new agents for preclinical testing (Tian et al. 2020; Hanson et al. 2018). More recently, rapid advances in iPSC and related technologies have provided human cell-based models of ALS that have identified molecules targeting hyperexcitability for pre-clinical development (Huang et al. 2021). The power of iPSC-based approaches to understanding disease and for identifying new therapeutics are becoming increasingly clear, and are exemplified by iPSC-based screens identifying small molecule drugs that have entered clinical trial for ALS (Morimoto et al. 2023). Here, we review how iPSC-based studies have contributed to the current knowledge of excitotoxicity in ALS, and explore novel approaches to inducing excitotoxicity in iPSC-derived neurons to further our understanding of its pathological pathways.

## Generating iPSC-Derived Neurons

iPSCs are somatic cells reprogrammed into an embryonic pluripotent state through the forced expression of the transcription factors Oct3/4, Sox2, c-Myc, and Klf4 in-vitro (Takahashi and Yamanaka 2006; Takahashi et al. 2007). The two most common somatic cell types used for pluripotent reprogramming are peripheral blood mononuclear cells (PBMCs) or fibroblasts collected through skin biopsy from human donors (Bardelli et al. 2020), enabling researchers to study disease pathology in a cell-specific, yet minimally invasive manner. The reprogramming process retains the DNA from the donor (Okano and Yamanaka 2014; Okuno et al. 2017). This provides researchers the ability to generate biological replicates by reprogramming iPSCs derived from different individuals, either with or without a specific phenotype. This feature of iPSC reprogramming also means that iPSCs can be generated directly from the somatic cells of patients and thereby enable study of disease mechanisms. However, during reprogramming, age-related epigenetic modifications revert to an embryonic state, which is a factor that needs to be considered for modelling age-associated diseases.

A major advantage to using iPSCs is the ability to differentiate them into virtually any cell type from the three primary germ layers, such as hepatocytes, smooth and skeletal muscle cells, and neurons (Dastouri et al. 2023; Talbot et al. 2021; Yiangou et al. 2018). Protocols have been developed to direct iPSC differentiation into general cell types, such as neurons, and to generate specific cell subtypes, for example cortical neurons or motor neurons (Barral and Kurian 2016; Sun et al. 2018; Yang et al. 2023). As ALS is characterised by upper and lower motor neuron pathology, where upper or lower motor neuron dysfunction is associated with clinical symptoms, disease severity, and survivability (Milella et al. 2023; Zakharova and Abramova 2022), there is also value in developing protocols to generate these two neuronal subtypes to be studied separately. A number of protocols for lower motor neuron differentiation have been published, however some challenges with directing iPSC differentiation into upper motor neuron cultures are yet to be overcome (Limone et al. 2023; Sances et al. 2016; Yang et al. 2023).

## Excitation in iPSC-Derived Neurons

**Fig. 2:**
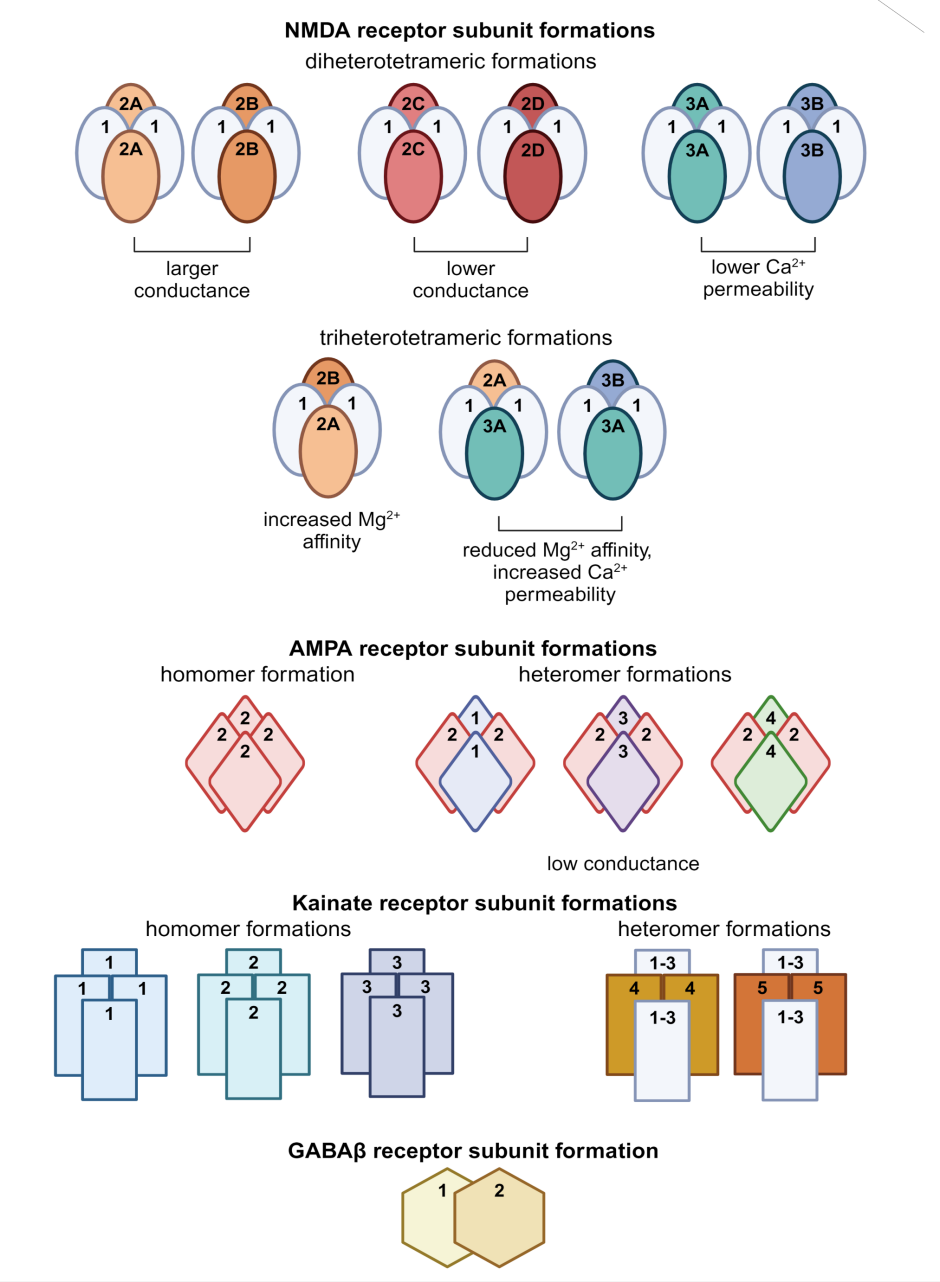
Schematic depicting subunit formations of NMDA, AMPA, KA, and GABA receptors. NMDA and AMPA receptor subunits dictate cellular properties including calcium permeability and receptor conductance, where subunit expression levels can alter neuron susceptibility to excitotoxicity (Cull-Candy and Leszkiewicz 2004; Herguedas et al. 2016). While KA receptors are understood to form homomers and heteromers, the properties of each receptor subunit are not fully known (Hansen et al. 2021; Watanabe-Iida et al. 2016)

**Fig. 3:**
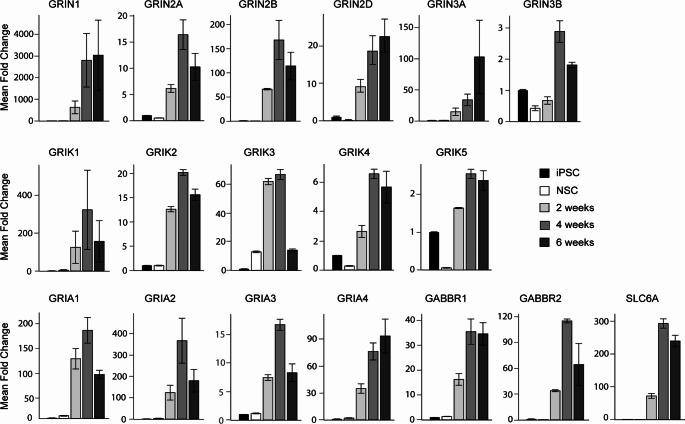
Gene expression levels of neurotransmitter receptor subunits in iPSC-derived cortical neurons. Human iPSC cell line TOB-00220 was maintained in mTeSR Plus medium, and differentatied to PAX6- and Nestin-positive neural stem cells (NSCs), that were then differentiated to MAP2- and TAU-postive neurons as we have described previously (Chear et al. 2022; Talbot et al. 2021). RNA was extracted and RT-qPCR performed using TaqMan probes and analysed according to Chear et al. (2022) to demonstrate levels of neurotransmitter receptor subunit mRNA expression in iPSCs, NSCs, and neurons at 2, 4, and 6 weeks maturation. NMDA, AMPA, KA, and GABA receptor subunits were upregulated in neurons compared to iPSCs and NSCs. *n* = 3 biological replicates, data presented as mean ± standard error of the mean. The TOB-00220 iPSC line used for these experiments was generated previously (Daniszewski et al. 2022), and verified using a 10-panel short-tandem repeat analysis in March 2022 using services from the Australian Genome Research Facility

**Fig. 4:**
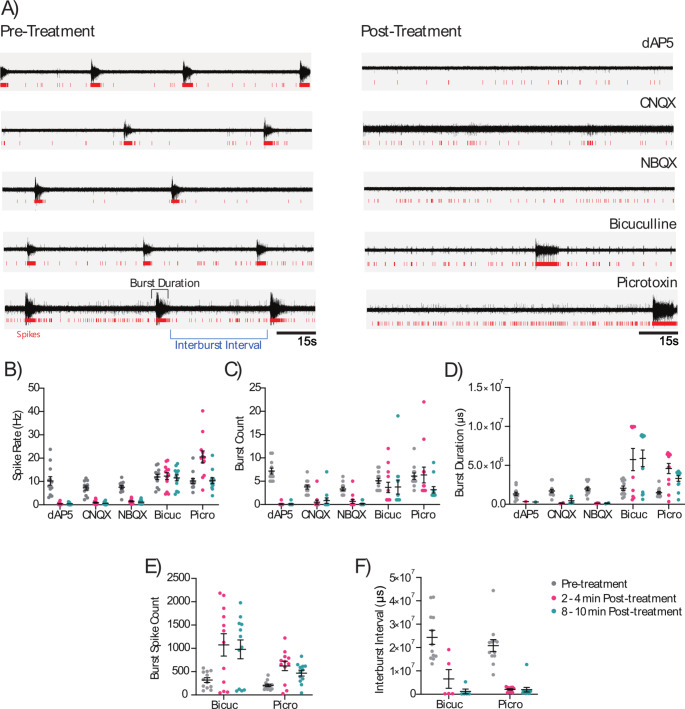
iPSC-derived cortical neurons express functional neurotransmitter receptors that can be modulated by known neurotransmitter antagonists. Human iPSC cell line TOB-00198 was maintained in mTeSR Plus medium, and differentatied to PAX6- and Nestin-positive neural stem cells (NSCs), that were then differentiated to MAP2- and TAU-postive neurons as we have described previously (Chear et al. 2022; Talbot et al. 2021). MEA recordings were obtained and analysed essentially as we have described previously (Chear et al. 2022), using 12-electrode 24-well plates from Multichannel Systems. Neuronal activity was recorded for 10 min at 37 °C, following a 2 min equilibration, and data processed using threshold parameters for determination of spikes, and bursts. Antagonists were applied during recording, by adding equal volume of medium with 2 × final concentration. **A**) iPSC-derived neurons exhibit spontaneous action potential firing, which was altered by treatment with NMDA (dAP5, 10 µM), AMPA (CNQX, 25 µM; NBQX, 10 µM), and GABA (bicuculline, 10 µM ; picrotoxin, 100 µM) antagonists. **B, C, D**) Application of dAP5, CNQX, and NBQX attenuated neuronal activity, through a reduced spike rate, burst count, and burst duration, while treatment with bicuculline and picrotoxin increased neuronal activity. **E, F**) Treatment of iPSC-derived neurons with bicuculline and picrotoxin increased burst spike count and decreased interburst interval. Circles represent values of individual electrodes; error bars represent mean and standard error. The TOB-00198 iPSC line used for these experiments was generated previously (Daniszewski et al. 2022), and verified using a 10-panel short-tandem repeat analysis in March 2022 using services from the Australian Genome Research Facility

A key consideration for modelling excitotoxicity is the maturity of the neurons produced from iPSCs. As described above, glutamate is a major excitatory neurotransmitter involved in excitotoxicity. Thus to model excitotoxicity, iPSC-derived neurons must express relevant glutamate receptor subunits and other proteins essential for neurotransmission, form functional receptors and other ion channels, and localise these at synapses to form neuronal networks. iPSC-derived neurons have been shown to express functional NMDA, non-NMDA (AMPA and KA), GABA, and glycine receptor subunits (Table 1; Fig. 2, and Fig. 3), which facilitate the actions of glutamate on the cell (Fig. 2), and are responsive to receptor agonists and antagonists that alter cellular activity (Fig. 4).

The vulnerability of neurons to excitotoxic mechanisms depends on NMDA and non-NMDA subunit composition within assembled neurotransmitter receptors (Vizi et al. 2013; Zhou and Baudry 2006; Zhou et al. 2013), and is attributed to differences in calcium permeability of each receptor (Armada-Moreira et al. 2020; von Engelhardt et al. 2007) (Fig. 2). Glutamate activity, and subsequent excitotoxicity, at NMDA receptors (NMDAR) is tightly controlled by the expression of the NMDA GluN2 receptor subunit, which provides the binding site for glutamate (Martel et al. 2012; Wyllie et al. 2013), whilst AMPA GluA2 receptor subunit expression creates calcium impermeable AMPA receptors (AMPAR) (Hollmann et al. 1991; Wright and Vissel 2012). AMPARs lacking the GluA2 subunit are highly permeable to calcium (Cull-Candy and Farrant 2021; Lalanne et al. 2018, Pál 2018), and contribute to excitotoxic cell death (Wright and Vissel 2012). Similar to AMPA receptors, KA receptor (KAR) calcium permeability is controlled by expression of the GluR2 subunit, which reduces cellular calcium permeability (Hollmann et al. 1991).

Functional NMDAR and AMPAR are expressed in neurons derived from human iPSCs, demonstrated through use of multiple techniques including calcium imaging, patch clamp, and multi electrode array (MEA) recordings (Antonov et al. 2019; Autar et al. 2022; Ishii et al. 2017). iPSC-derived cortical neurons express the GRIN1, GRIN2A and GRIN2B genes, which encode the NMDAR GluN1, GluN2A, and GluN2B subunits, respectively (Antonov et al. 2019; Klima et al. 2021). Application of glutamate, NMDA, AMPA and KA to iPSC-derived neuronal cultures increases the activity (spike frequency) of neurons, suggesting that iPSC-derived neurons express functional NMDA and non-NMDA receptors (Klima et al. 2021; Odawara et al. 2016). Additionally, antagonism of NMDA and non-NMDA receptors on iPSC derived neurons, attenuates neuronal functional activity confirming the contribution of these receptors to neuronal firing (Odawara et al. 2016; Autar et al. 2022). Functional NMDAR subunits, GluN1, GluN2A and GluN2B are also expressed in iPSC-derived brain organoid models facilitating neuronal activity (Bauersachs et al. 2021). The NMDAR subunit expression in iPSC derived neurons varies with cell type where iPSCs in mixed cortical neuronal culture have been reported to predominantly express GluN2B subunits over GluN2A subunits (Zhang et al. 2016), whilst iPSCs differentiated into ventral mesencephalic neurons, have a higher expression of GluN2A subunits (Antonov et al., 2019).

iPSC-derived cortical neurons from healthy adult donors, showed expression of NMDA, AMPA, KA, and GABA receptor subunits using reverse transcription qPCR techniques (Fig. 3). These iPSC-derived cortical neurons recorded on MEA plates and analysed according to Chear et al. (2022), were electrically active, firing trains of spontaneous action potentials (Fig. 4), confirming their functionality. Application of NMDA (dAP5), AMPA (CNQX, NBQX) and GABA (bicuculline and picrotoxin) antagonists, altered the firing properties of these iPSC-derived cortical neurons, affirming the presence of functional receptors in these cells. Application of dAP5, CNQX and NBQX attenuated neuronal firing, decreasing neuronal spike rate and burst firing dynamics (properties) (Fig. 4B, C, and D) compared to pre-treatment activity. Application of bicuculline and picrotoxin, minimally changed spike rate but altered burst firing dynamics, increasing the number of spikes per burst and decreasing inter-burst intervals (Fig. 4E and F). This activity pattern likely derives from a mixed iPSC-derived cortical network of both excitatory and inhibitory neurons where the ratio of excitatory neurons is likely greater than inhibitory neurons.

**Table 1:** Neurotransmitter receptor subunits and corresponding gene names

Receptor type	Subunit name	Gene name
NMDA	GluN1	*GRIN1*
	GluN2A	*GRIN2A*
	GluN2B	*GRIN2B*
	GluN2D	*GRIN2D*
	GluN3A	*GRIN3A*
	GluN3B	*GRIN3A*
AMPA	GluA1	*GRIA1*
	GluA2	*GRIA2*
	GluA3	*GRIA3*
	GluA4	*GRIA4*
KA	GluK1	*GRIK1*
	GluK2	*GRIK2*
	GluK3	*GRIK3*
	GluK4	*GRIK4*
	GluK5	*GRIK5*
GABA	GABA_B1_	*GABBR1*
	GABA_B2_	*GABBR2*
	GAT1	*SLC6A1*

## Modelling Excitotoxicity in iPSC-Derived Neurons

### Excitotoxicity in iPSC-Derived Genetic Disease Models

ALS symptom onset can arise through familial inheritance of ALS-associated causative genes, the most common being mutations in the superoxide dismutase 1 (SOD1), C9ORF72, TAR DNA binding protein (TARDBP), and fused-in-sarcoma (FUS) genes. Thus, transgenic animal models carrying these major causative genes have been generated and used extensively in ALS studies, including ALS-associated excitotoxicity. However, there are limitations to modelling disease in animals, such as mice or Drosophila, given the phylogenetic differences between humans and other species. These limitations are evident in the low success rates in translating promising drug treatment in SOD1 mouse models to clinical cases, despite being a widely used model because of its recapitulation of pathological and functional changes in ALS (Achilli et al. 2005). Other ALS-associated genes, such as the C9ORF72, TARDBP, and FUS genes have also been studied in many animal models but still present challenges in fully mimicking ALS pathology (Morrice et al. 2018; Amalyan et al. 2022; O’Rourke et al., 2015; Peters et al. 2015).

A key advantage in using iPSCs compared to other preclinical models is the ability to generate patient-derived cell lines, where gene variants contributing to ALS development are preserved. Notably, the majority of ALS cases develop sporadically and may not feature variants within genes identified in familial ALS cases, or may feature a combination of genetic factors that increase disease risk. Several iPSC models based on variants within the major genes associated with ALS have revealed electrophysiological relevance to ALS. For example, evoked action potential frequency was higher in C9ORF72^HRE^ motor neurons compared to controls (Burley et al. 2022). SOD1^G93A^ motor neurons have a longer burst duration compared to isogenic controls, although other parameters including network bursting were decreased (Kim et al. 2020). Other findings include increased firing frequency in TDP-43^G298S^ and C9ORF72^HRE^ motor neurons (Harley et al. 2023), and increased firing rate in SOD1^A5V^ neurons (Wainger et al. 2014). iPSC models of ALS have also revealed changes to the axon initial segment (Harley et al. 2023; Lefebvre-Omar et al. 2023), a compartment of the base of the axon responsible for action potential generation and propagation (Leterrier 2018).

Given the expression of NMDAR and non-NMDAR in iPSC-derived neurons, iPSC-derived from patients with neurodegenerative-linked genetic mutations can be used to investigate the vulnerability, selectivity and sensitivity of neurons to excitotoxic mechanisms that are pathological hallmarks of ALS. Dysregulation of AMPA and KA receptor subunits are associated with increased non-NMDAR calcium permeability and contributes to excitotoxicity of motor neurons in ALS. Motor neurons with ALS-linked mutations derived from patients, are sensitive to excitotoxicity as they combine an inability to buffer calcium with an increase in AMPAR expression and calcium permeability (Van Den Bosch et al. 2006), resulting in increased intracellular calcium (Bursch et al. 2019; Dafinca et al. 2020; Selvaraj et al. 2018). iPSC-derived motor neurons cultured from ALS patients carrying mutations in the C9ORF72 gene, significantly upregulated calcium permeable AMPAR (GluA1 and GluA3) and NMDAR subunits, causing a persistent expression of inward calcium currents, higher intracellular calcium, and altered calcium clearance and buffering, leading to increased vulnerability of these motor neurons to excitotoxicity and subsequent neural death (Dafinca et al. 2020; Selvaraj et al. 2018). Selectively blocking calcium permeable AMPARs in iPSC-derived mutant C9ORF72 motor neurons reduced cell death (Selvaraj et al. 2018), whilst CRISPR/CAS9-corrected gene editing of C9ORF72 iPSC-derived motor neurons abolished calcium dysregulation in these neurons (Dafinca et al. 2020; Selvaraj et al. 2018). Similar to motor neurons carrying C9ORF72 mutations, iPSC-derived motor neurons from patients carrying TARDBP mutations had altered (delayed) calcium buffering, increased expression of calcium permeable AMPA (GluA4) and NMDA receptor subunits (GluN1 and GluN2B) (Dafinca et al. 2020), and elevated basal intracellular calcium levels (Bursch et al. 2019). iPSC-derived motor neurons from patients with mutations in SOD1 had elevated expression of KAR, voltage-gated calcium channels and metabotropic glutamate receptors, and mutant FUS iPSC derived motor neurons had increased AMPAR and KAR expression (Bursch et al. 2019).

### KA-Induced Excitotoxicity

Excitotoxicity is observed across ALS cases and is reflected in iPSC-derived genetic models, despite the heterogeneity in underlying genetic causes. Thus, one strategy to understanding pathology is to focus on the mechanisms and pathways of excitotoxicity-induced neurodegeneration and model excitotoxicity in the absence of genetic mutations. Experimentally, excitotoxicity can be induced through application of glutamate or a glutamate agonist like KA, which drive hyperexcitability, an increased capacity of a neuron to respond to a stimuli. Excessive stimulation of glutamate receptors on human embryonic stem cell (hESC)-derived neurons, through the application of glutamate or glutamatergic analogues, induces neuronal hyperexcitability which coincides with the developmental expression of NMDAR and non-NMDAR subunits (Gupta et al. 2013). In iPSC-derived forebrain 3D organoid models, glutamate-induced excitotoxicity is mediated by NMDARs, as application of glutamate or NMDA caused stereotypical excitotoxicity pathology including morphological and structural neuronal changes, neurite blebbing and cell death (Bauersachs et al. 2021). This increased functional hyperactivity and subsequent cell death in both 2D and 3D models, can be blocked by NMDAR antagonists, highlighting a central role for NMDA-induced excitotoxic neuronal dysfunction and death (Bauersachs et al. 2021; Gupta et al. 2013).

KA, a nondegradable analog of glutamate, is a potent agonist of AMPA and KA receptors and therefore a potent neurotoxin (Vincent and Mulle 2009). The neural cell death pathway induced by KA induces apoptosis and necrosis similarly to prolonged glutamate activity (Wang et al. 2005). KA bound to AMPAR and KAR induces rapid cellular Ca^2+^ influx, which activates Ca^2+^ dependent enzymes and triggers decreased ATP production and the generation of reactive O_2_ species (ROS), which leads to oxidative DNA and mitochondrial damage and dysfunction (Wang et al. 2005). The pathological changes induced by KA partially mimic neurodegeneration (Wang et al. 2005; Zheng et al. 2011) and thus KA has emerged as a robust model of inducing excitotoxicity.

Much of what we understand about KA-linked excitotoxicity, particularly KA-linked axon degeneration, has come from rodent primary cultures that rely on visualization of neuron morphology to quantify the degree of degenerative damage within the neuron. In response to KA application, cultured mouse motor neurons developed focal neurofilament swellings and neuronal beading within the axon in a dose dependent manner, where axonal degeneration worsens with increasing KA concentration and exposure (King et al. 2007). KA induced axonopathy and degeneration in cultured neurons precedes neuronal death and is triggered largely by the activation of non-NMDA receptors, although NMDA receptors can contribute (King et al. 2007). Changes to the developmental expression of NMDA and non-NMDA receptor subunits on cultured neurons coincide with their selective vulnerability to excitotoxicity. Immature cultured neurons are not vulnerable to KA toxicity or distal axonal swelling (Gupta et al. 2013; King et al. 2006), however, their susceptibility to excitotoxicity-linked degeneration increases with neuronal maturation, synapse formation and glutamate receptor subunit expression (King et al. 2006).

KA excitotoxicity induced neurodegeneration in human iPSC-derived neurons has recently been studied. iPSC-derived neurons treated with low (6.25µM) concentrations of KA for 24 h showed no morphological changes. However, increasing concentrations of KA caused degenerative changes in neuronal morphology, including axon fragmentation and beading, where high KA concentrations caused complete degeneration of all neuronal processes (Talbot et al. 2021). KA treatment on mature (68 days in vitro) iPSC-derived cortical neurons for 24 h acutely increased neuronal bursting activity, which then subsequently decreased 24 h post KA application (Mzezewa et al. 2022). Other studies investigating excitotoxicity linked neurodegeneration have used human iPSC-derived glutamatergic neurons (iCell^®^ GlutaNeurons) and investigated their functional activity in response to glutamate agonists including kainic acid (Chen 2018). KA application over 24 h increased neuronal mean firing rate and caused concentration-dependent reductions in cell viability (cell density), highlighting successful KA-induced toxicity (Chen 2018).

### Inducing Excitotoxicity through Chemogenetics

Another approach to increasing excitation within iPSC-derived neuronal cultures is through the application of chemogenetics. Chemogenetics is defined as the engineering of protein receptors to respond to previously unrecognised small molecule ligands, where these protein receptors can then be expressed in targeted cell populations (Forkmann and Dangelmayr 1980; Sternson and Roth 2014; Strobel 1998). These protein receptors are engineered to be inert with ligands native to the cell environment and recognised ligands are selected to not affect endogenous signalling, allowing for specific control over a targeted cell population expressing chemogenetic receptors. Genes encoding the receptors can be delivered into the target cell population through viral vectors, and insertion into the genome through CRISPR/Cas technology (Kawai et al. 2021; Wang et al. 2013; Xiong et al. 2021). Thus, chemogenetics are a powerful tool allowing researchers to activate chemogenetic receptor expressing neurons in a controlled manner and exploring the effects of hyperexcitability on the neuron.

**Fig. 5:**
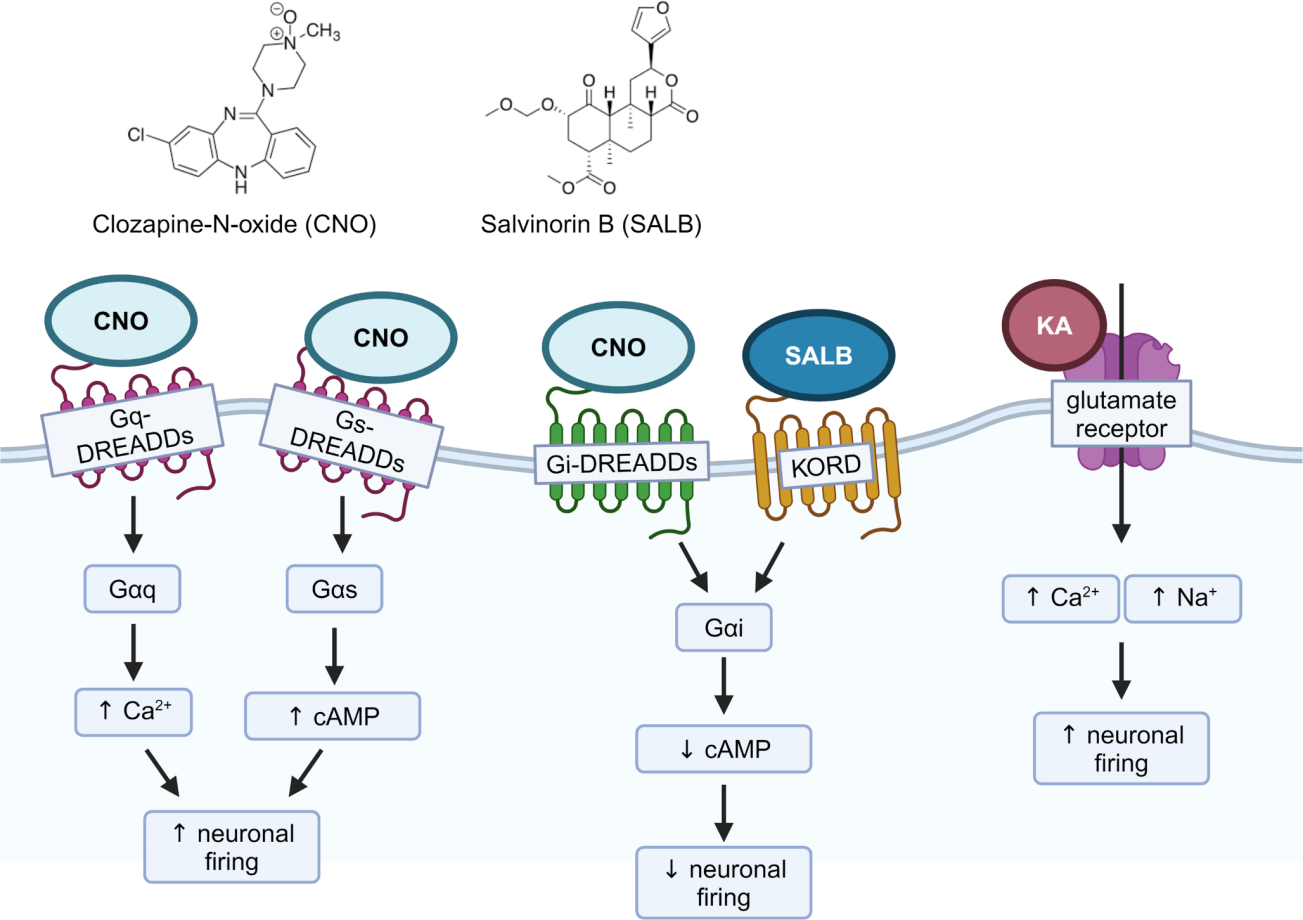
Modulation of neuronal activity using DREADDs. DREADDs can be expressed in neurons to increase or decrease neuronal firing, depending on the subclass of DREADD expressed. DREADDs are designed to bind specifically with ligands not typically found in the natural environment of neurons, such as CNO and SALB. DREADDs, while derived from human muscarinic receptors, cannot interact with endogenously found neurotransmitters such as glutamate or other ligands, allowing for specific control of neuronal activity

The most widely used class of chemogenetically engineered protein receptors are Designer Receptors Exclusively Activated by Designer Drugs (DREADDs), which are based on engineered G protein-coupled receptors (GPCRs) (Armbruster and Roth 2005; Armbruster et al. 2007; Roth 2016) (Fig. 5). The classification of DREADD types is based on the corresponding protein each engineered GPCR interacts with, and each DREADD class can either activate or inhibit neurons through downstream effects on cell function (Alexander et al. 2009; Farrell et al. 2013; Vardy et al. 2015; Zhu et al. 2014). Gq-DREADDs are based on human muscarinic receptors and signal through Gαq/11 protein, activating neuronal firing via ligands such as clozapine N-oxide (CNO) (Alexander et al. 2009), whilst Gs-DREADDs increase neuronal activity, through CNO-dependent Gαs protein activity (Farrell et al. 2013), and thus can be used to mimic cellular excitability changes seen in degenerative diseases. Conversely, DREADD classes that can inhibit neuronal signalling through inhibition of downstream cAMP production are referred to as Gi-DREADDs, signalling through the Gαi/o protein or the human κ-opioid receptor, and bind to CNO and salvinorin B (SALB), respectively (Vardy et al. 2015; Zhu et al. 2014).

The most commonly employed DREADD for increasing neuronal firing is the Gq-DREADD called hM3Dq, which is activated using CNO (Alexander et al. 2009). While there are currently limited human iPSC-based DREADD studies, in vivo rodent models have demonstrated that selective activation of targeted neuronal subtypes can result in modulated behaviour, such as induced hyperactivity or enhanced memory function (Shibano et al. 2020; Wang et al. 2013). Furthermore, hM3Dq expressed from the calcium/calmodulin dependent protein kinase II-α (CAMKIIα) promoter can be activated to induce ALS-like motor deficits, as well as corticomotoneuron and spinal motor neuron loss in mice (Haidar et al. 2021). In human iPSC-derived neurons where hM3Dq is expressed under control of the CAG promoter, CNO-induced activation leads to increased intracellular Ca^2+^ levels (Kawai et al. 2021). When transplanted into injured mouse spinal cords, activated hM3Dq-expressing human iPSC-derived neurons alter synapse related gene expression levels in the neighbouring non-DREADD expressing mouse neural cells. The increased intracellular Ca^2+^ levels and modulation of synapse related gene expression indicates an increased excitability in cells and resemble changes seen in ALS-derived neurons, suggesting that DREADDS can be employed as a potential method of inducing hyperexcitability or excitotoxicity in iPSC-derived neurons in the absence of ALS-associated gene mutations.

Rodent studies also demonstrate that DREADD-expressing neurons respond to CNO treatment in a dose-dependent manner, where higher concentrations of CNO lead to increased neural activity as well as long term changes to the neural circuit (Pati et al. 2019; Wang et al. 2013). Therefore, there is potential for using DREADDs as a tool for inducing excitotoxicity in hiPSC-derived neurons through treatments either with high concentrations of CNO or long-term, lower doses of CNO to compare the effects of acute and chronic hyperexcitability on neuronal health.

Experimentally inducing excitotoxicity through the activation of DREADDs can be complementary to studying KA-induced excitotoxicity as the two approaches increase neuronal activity through separate signalling pathways (Fig. 5). KA modulates neuronal activity primarily through ionotropic glutamate receptors and their associated downstream pathways, while DREADDs, being derived from G-protein coupled receptors, more closely mimic the activity of metabotropic receptors. Therefore, comparison between the two approaches would lead to a more comprehensive understanding of the downstream mechanisms resulting in excitotoxicity-induced neurodegeneration as well as assist in identifying potential therapeutic targets for ALS.

### Excitotoxicity as a Consequence of ALS-Associated Gene-Environment Interactions

ALS symptom onset is hypothesized to be a multi-step process, resulting from the interaction between inherited genetic factors and the sum of environmental exposures throughout a person’s life, which has been referred to as the exposome (Al-Chalabi et al. 2014; Goutman et al. 2023). This has presented a challenge for accurately modelling ALS, and consequently hindering identification of effective therapeutic targets. Some ALS-associated environmental factors are suggested to have excitotoxic effects. One such example is chlorpyrifos, a type of organophosphate pesticide. Organophosphates are known to cause neuropathy following acute, high-dose exposure, and there is evidence suggesting an increased risk of ALS with long-term, low-dose organophosphate exposure (Merwin et al. 2017; Andrew et al. 2021). As an organophosphate pesticide, the key neurotoxic mechanism of chlorpyrifos is the irreversible inhibition of acetylcholinesterase (AChE), which leads to the accumulation of acetylcholine (ACh) at the synapses and the overstimulation of cholinergic and muscarinic receptors (Pulkrabkova et al. 2023). However, evidence suggests that chlorpyrifos can also lead to glutamate-mediated hyperexcitability (Fig. 1). It is hypothesised that permanent stimulation of muscarinic cholinergic receptors resulting from chlorpyrifos exposure can activate the glutamatergic system through activation of ionotropic glutamate receptors (Pulkrabkova et al. 2023). Early studies show that chlorpyrifos exposure in mouse cortical cell cultures leads to increased levels of extracellular glutamate, and that chlorpyrifos-induced neurotoxicity can be attenuated by glutamate receptor antagonists (Rush et al. 2010). Treatment with otherwise sub-lethal dose of chlorpyrifos is also shown to increase susceptibility to glutamate toxicity in mature mouse neuronal cultures, suggesting that low-dose exposure to chlorpyrifos may affect the vulnerability of neurons to additional excitotoxic insults (Amani et al. 2016).

The cyanotoxin beta-methylamino-L-alanine (L-BMAA) has also been implicated in the development of sporadic ALS (Kisby and Spencer 2011; Goutman et al. 2023). While neurotoxic mechanisms of L-BMAA are still unknown, there is early evidence that it acts through interacting with NMDA and AMPA/KA receptors (Delcourt et al. 2017; Lobner et al. 2007; Rao et al. 2006). Studies in rodent models have shown that L-BMAA exposure can lead to excitotoxicity, however only in concentrations higher than are seen in cases of L-BMAA related ALS (Duncan et al. 1991; Okle et al. 2013). Chronic exposure to sub-excitotoxic levels of L-BMAA have been shown to result in cell dysfunction (Burton et al. 2023), however neuronal activity and hyperexcitability is yet to be explored in low-dose L-BMAA exposure studies. iPSC-derived neurons can provide a platform to explore this gap of knowledge, exploring the effects of low-dose L-BMAA on neuronal activity. Additionally, mutant TARDBP mouse motor neurons are shown to be more vulnerable to L-BMAA-induced dysfunction than wild type (Arnold et al. 2023), supporting the concept of gene-environmental interactions leading to ALS and iPSC-derived neurons can provide further insight on the electrophysiological effects of combining ALS causative genes with environmental factors.

## Measuring Neuronal Activity in Hyperexcitability Models: Patch Clamping vs. MEAs

Electrophysiological techniques have been developed to manipulate the electrical activity of neuronal populations, with single cell and population-based configurations employed as reliable, flexible and adaptive ways to understand intrinsic and network based iPSC neuronal activity.

Intrinsic activity in iPSC-derived neurons is routinely investigated using single cell electrophysiological approaches, which offer accurate single-cell resolution within an in vitro neuronal population (Table 2). Patch clamp techniques have been used in iPSC-derived neurons for the characterisation of intrinsic passive cellular properties, including membrane potential and membrane resistance, and active cellular properties, like firing rate and spontaneous activity (Halliwell et al. 2021; Jezierski et al. 2021). Together, the electrophysiological properties of iPSC-derived neurons can be used to characterise and classify neuronal cell types, monitor the maturity and degree of differentiation of iPSC-derived neurons and allow comparisons between cell lines (Halliwell et al. 2021; Jezierski et al. 2021; Vahsen et al. 2022; Smith et al. 2021; Taga et al. 2019). Patch clamp techniques allow for the manipulation of cellular membrane properties, firing pattern and synaptic activity (Devlin et al. 2015), where agonists and antagonists of glutamate and GABAergic/glycinergic receptors selectively isolate iPSC-derived neuron ionic currents, whilst application of neuro-active drugs can determine the specific neuropharmalogical profile of a population (Halliwell et al. 2021; Jezierski et al. 2021).

Neuronal activity is nuanced, and hyperexcitabiliy may manifest as changes in more than one electrophysiological parameter, whereby employing a multi-parameter readout provides a holistic measure of cell activity. In ALS, patch clamp recordings of C9ORF72 iPSC-derived motor neurons showed evoked action potential firing increased in C9ORF72 neurons compared to control neurons, which was independent of fast inactivating or delayed rectifier K^+^ channels (Burley et al. 2022). iPSC-derived motor neurons from ALS patients carrying either a TARDBP or C9ORF72 mutation were hyperexcitable compared to control motor neurons where the number of cells firing functional action potentials significantly decreased over time (Devlin et al. 2015). Patch clamped iPSC-derived motor neurons with mutant TDP-43^Q331K^ expression had reduced repetitive firing but an increased resting membrane potential in mutant cells when compared to control cells, indicating that mutant TDP-43^Q331K^ motor neurons are primed to respond to less input from surrounding cells (Smith et al. 2021).

The advantage of patch clamp electrophysiology is the ability to understand the functional activity of neurons at a single cell resolution. However, using patch clamp techniques to investigate the activity of a neuronal population or network can be labourious and time-consuming. Multielectrode array (MEA) techniques offer a non-invasive method to record extracellular activity from neural populations, capturing population-based activity dynamics including network synchronisation (Mossink et al. 2021). iPSC-derived neurons are grown on custom MEA well plates, where electrodes embedded in the base of each well enable the extracellular recording of action potentials attributable to the cells directly on top of the electrodes, and are now tailored to record population activity with subcellular resolution (Ronchi et al. 2021).

The use of in vitro MEA techniques for functional disease phenotyping of iPSC-derived neuronal cultures continues to increase in popularity, in part due to its high-throughput design, spatiotemporal resolution and ability to interrogate population-wide functional activity in a number of modelled disease states (Table 2). In addition to standard MEA plates that record functional network dynamics, high-density-MEAs (HD-MEAs) offer subcellular electrical activity resolution that can track action-potential propagation velocities across multiple electrodes (Ronchi et al. 2021; Buccino et al. 2022). Unlike patch clamp techniques, which are mechanically invasive to the cell, the non-invasive nature of MEA allows cultured cells to be repeatedly recorded for months to temporarily monitor functional activity across development, or throughout disease trajectories (Ronchi et al. 2021). In ALS, MEA techniques have been used to characterise and compare hiPSC lines carrying ALS-linked mutations, where motor neurons derived from ALS patients (hMN-ALS) (Ronchi et al. 2021) or iPSC-derived motor neurons carrying a SOD1^G93A^ missense mutation (Kim et al. 2020) had lower mean firing rates, more sporadic firing, and lower number of network bursts compared to control neurons (Kim et al. 2020; Ronchi et al. 2021).

A combinatorial approach using both MEA and patch clamp techniques, has been used to characterise the intrinsic and population-based activity of iPSC neurons derived from ALS patients, or carrying induced ALS mutations (Table 2). Although MEAs can quantify network-level activity more readily than patch-clamping, the two methods provide complementary, and congruent results across different ALS models. MEA recordings of ALS-derived iPSC motor neurons carrying a SOD1^A5V^ allele had increased spontaneous action potentials and higher mean firing rate, likely due to increased intrinsic excitability (Wainger et al. 2014). Single-cell recordings from these SOD1^A5V^ motor neurons confirmed a reduction in the delayed rectified potassium channel, which is responsible for membrane repolarisation to baseline, thus increasing excitability in these cells (Wainger et al. 2014). Sporadic ALS iPSC-derived cervical spinal motor neurons, showed significant spontaneous electrical activity (spikes) characterised by an increase in spontaneous spikes, bursts and network bursts compared to control spinal motor neurons, which was confirmed with patch clamp recordings (Yang et al. 2023). In iPSC-derived motor neurons with mutant TDP-43^Q331K^, an increased resting membrane potential combined with a reduction in repetitive firing pattern recorded through patch clamp electrophysiology, was matched at a population level through MEA recordings by a notable decrease in burst firing (Smith et al. 2021). Therefore patch clamp and MEA techniques, when used in concert, provide complementary data regarding neuronal functional phenotype(s) and network properties of a neuronal population, and thereby provide insight into how electrophysiology might be altered in iPSC-derived ALS disease models.

**Table 2:** iPSC-based studies reporting electrophysiology

Paper	Disease/Mutation	Neuronal Subtype	Electrophysiological Technique/s	Drug Treatment	Selective Outcomes
**ALS – C9ORF72 iPSC lines**					
Burley et al. (2022)	ALS (C9ORF72)	Motor neurons	Whole-cell patch clamp		C9ORF72 MNs C_m_, R_m_, RMP = control. C9ORF72 MNs ↑ firing frequency @ 40 DIV. *I*Na^+^ and *I*K^+^ density = control.
Devlin et al. (2015)	ALS patient-derived lines (TARDBP, C9ORF72)	Motor neurons	Whole-cell patch clamp		C9ORF72 iPSC-derived MNs ↓ C_m_ compared to TARDBP MNs, TARDBP MNs ↑ C_m_ compared to control MNs. R_in_ = across cell lines. C9ORF MNs ↑ RMP, ↑ FF compared to control. C9ORF MNs firing ↓ over time. C9ORF72 MNs had glutamate, GABA + glycine currents, C9ORF72 MNs ↓ synaptic activity over time. C9ORF72 MNs ↓ *I*Na^+^ + *I*K^+^ over time.
Wainger et al. (2014)	ALS, SOD1^A5V^ and C9ORF72 from familial ALS patients	Motor neurons	MEA Whole-cell patch clamp	Retigabine, bicuculline, strychine, dAP5, CNQX, flupirtine, TTX	MEA: Retigabine + flupirtine ↓ spontaneous APs in C9ORF72 MNs. Patch clamp: ALS MNs ↑ AP number compared to control. RMP, AP_thres_, R_in_ = between ALS and control MNs. Delayed rectifier *I*K^+^ ↓ in mutant MNs. C9ORF72 MNs from ↑ MFR compared to controls.
**ALS – SOD1 iPSC lines**					
Huang et al. (2021)	ALS (SOD1 *A5V*)	Motor neurons	MEA Whole cell patch clamp		SOD1^A5V^ MNs have spontaneous firing, FF ↑ with maturation, firing activity ↓ with AMPA receptor and *I*K^+^ antagonists. AMPA ↑ FF
Kim et al. (2020)	ALS (SOD1 *G93A* and *A5V*)	Motor neurons	MEA Whole-cell patch clamp	TTX	SOD1 mutant iPSC MNs ↓ spontaneous APs, ↓ MFR, ↓ N^o^ network bursts, ↓ NBF compared to control MNs. SOD1 mutant MNs ↑ burst firing once network bursting was engaged, ↑ N^o^ spike/burst compared to control. TTX sensitive channels.
Wainger et al. (2014)	ALS, SOD1^A5V^ and C9ORF72 from familial ASL patients	Motor neurons	MEA Whole-cell patch clamp	Retigabine, bicuculline, strychine, dAP5, CNQX, flupirtine, TTX	MEA:↑ spontaneous APs + ↑ MFR in SOD1^A5V^ mutant MNs compared to control. Bicuculline + strychnine ≠ FR. SOD1^A5V^ corrected MNs ↓ spontaneous FR compared to uncorrected mutant MNs. Retigabine + flupirtine ↓ spontaneous APs in SOD1 MNs. Patch clamp: ALS MNs ↑ AP number compared to control. RMP, AP_thres_, R_in_ = between ALS and control MNs. Delayed rectifier *I*K^+^ ↓ in mutant MNs.
**ALS – TARDBP iPSC lines**					
Devlin et al. (2015)	ALS patient-derived lines (TARDBP, C9ORF72)	Motor neurons	Whole-cell patch clamp		TARDBP MNs ↑ C_m_ compared to control MNs. R_in_ = across cell lines. TARDBP MNs ↑ RMP, ↑ FF compared to control. N^o^ TARDBP firing ↓ over time. TARDBP MNs had glutamate, GABA + glycine currents; TARDBP + MNs ↓ synaptic activity over time. TARDBP MNs ↓ *I*Na^+^ + *I*K^+^ over time.
Smith et al. (2021)	ALS (TDP-43 *Q331K* and *M337V*)	Motor neurons; CDI TDP-43 mutant lines (Q331K and M337V) compared to isogenic control (CDI: R1049) WTC-11 heterozygous TDP-43 Q331K edited in-house and compared to wild type as isogenic control	MEA Whole-cell patch clamp,		MEA: CDI TDP43^M337V^ + TDP43^Q331K^ MNs ↓ burst firing, ↑ burst duration and weighted MFR compared to isogenic control. WTC11-derived MNs ↓ burst firing, ↓ burst duration in TDP43^Q331K^ MNs compared to isogenic control. RMP, FF, burst duration, bursts per minute = control. Patch clamp: TDP43^Q331K^ WTC11 MNs ↓ AP firing @90DIV and TDP43^M337V^ + TDP43^Q331K^ motor neurons ↑ RMP compared to control.
Ronchi et al. (2021)	ALS (TDP-43 Q331K), PD (a-synuclein A53T mutant)	Motor neurons (ALS), Dopaminergic neurons (PD)	CMOS-based HD-MEA	Retigabine	hMN-ALS cells ↓ MFR, ↓ regular firing, ↑ burst duration, ↑ interburst interval, ↓ burst/minute, ↑ axonal AP propagation velocity compared to control. Retigabine ↓ MN-ALS spontaneous activity compared to control.
**Other diseases**					
Mossink et al. (2021)	Kleefstra syndrome (KS), mitochondrial encephalopathy, lactic acidosis, and stroke-like episodes (MELAS)	Excitatory cortical neurons	MEA		Firing activity stabilised from 27 DIV, spontaneous activity, network bursting rate and network burst duration stable across control cell lines, network FR more variable across cell lines.
Ronchi et al. (2021)	PD (a-synuclein A53T mutant), ALS (TDP-43 Q331K)	Dopaminergic neurons (PD), Motor neurons (ALS)	CMOS-based HD-MEA	Retigabine	Dopaminergic -PD neurons ↓ MFR, ↓ burst duration, ↑ interburst interval compared to control at DIV 21. No change in axonal AP propagation velocity.
**Healthy iPSC lines**					
Autar et al. (2022)	Healthy	iPSC-derived cortical neurons	MEA Whole-cell patch clamp		MEA: Spontaneous firing ↓ by lidocaine, Glutamate ↑ FF, NBQX ↓ FF, Picrotoxin and bicuculline ↑ FF. Patch Clamp: Developmental ↑ in spontaneous and repetitive firing .
Bauersachs et al. (2021)	Healthy	Forebrain organoid	Whole-cell patch clamp	Lidocaine, NBQX, Picrotoxin, Bicuculline	Electrically active with AMPA and GABA currents.
Bianchi et al. (2018)	Healthy	iPSC-derived Motor Neurons	Whole-cell patch clamp		↑ electrical activity @21 DIV. Maturing iPSC-derived MNs showed APs and AP train firing.
Halliwell et al. (2021)	Healthy	iCell neurons (Fujifilm Cellular Dynamics Inc.)	MEA Whole-cell patch clamp	bicuculline, picrotoxin, chlordiazepoxide, diazepam, mefenamic acid, phenobarbital, allopregnanolone, glycine, AP5, DNQX,	MEA: Spontaneous activity from 48 h plating, TTX ↓ spike count. Patch Clamp: Developmental ↑ in spontaneous APs, AP_thresh_, C_m_, R_in_ and R_s_. TTX sensitive *I*Na^+^ and TEA sensitive *I*K^+^. Diazepam, chlordiazepoxide, allopregnanolone, mefenamic acid ↑ *I*GABA currents; picrotoxin and bicuculline ↓ *I*GABA; Glycine ↑ and strychnine ↓ *I*glycine; Kynurenic acid and kainic acid ↑ *I*AMPA, DNQX ↓ *I*AMPA; AP5 ↓ *I*NMDA.
Jezierski et al. (2021)	Healthy	CNS.4U (Ncardia, Gosselies), amniotic fluid-iPSC derived forebrain neurons (AF-iNs); mixed GABAergic, glutamatergic, and dopaminergic neurons	MEA Whole-cell Patch clamp		MEA: AF-iNs, Developmental ↑ in spontaneous activity, GABA ↓ spontaneous activity. Patch Clamp: AF-iNs repetitive AP firing, spontaneous activity and *I*_h_.
Nimtz et al. (2020)	Healthy	iPSC induced human neuronal networks (hNN)	MEA	GABA, bicuculline, glutamate, AP5, NBQX, DA	hNN had spontaneous activity, GABA ↓ MFR, MBR and N^o^ spikes/burst. Bicuculline ↓ MFR and N^o^ spikes/burst. AP5 and NBQX ≠ on hNN. DA ↑ spontaneous activity.
Odawara et al. (2016)	Healthy	hiPSC- derived cerebral cortical neurons	MEA	Bicuculline, KA, CNQX, AP5.	Spontaneous FF and spontaneous SBF ↑ over time. Bicuculline and KA ↑ spontaneous firing rate. CNQX ↓ spontaneous firing rate and abolished SBFs, AP5 ↓ SBF duration
Taga et al. (2019)	Healthy	hiPSC-derived motor neurons (MNs) cocultured with/without astrocytes	MEA	KA, CNQX, Bicuculline, KCl	Astrocyte co-cultured hiPSC-MNs ↑ spike frequency, ↑ burst rate,↑ N^o^ spikes than MNs co-culture alone. KA ↑ and CNQX ↓co-culture spike frequency. Bicuculline ≠. KCL ↑ overall depolarisation.
Vahsen et al. (2022)	Healthy	hiPSC-derived motor neurons with and without microglia co-culture	Whole-cell patch clamp		RMP, *I*Na^+^, *I*K^+^ and evoked APs similar between MN in monoculture and co-culture.

**FF** firing frequency, **FR** Firing rate, **C**_**m**_ membrane capacitance, **R**_**in**_ membrane resistance, **RMP** Resting Membrane Potential, **Rs** Series Resistance, **AP** Action potential, **AP**_**thresh**_ Action potential Threshold, ***I*** Current, **N**^**o**^ Number of, **DA** Domoic Acid, **KA** Kainic Acid, **DIV** Days in vitro, **MFR** mean firing rate, **MBR** mean bursting rate, **SBF** bynchronised burst firing, **NBF** network burst frequency, **TTX** Tetrodotoxin, **↓** Decreased, **↑** Increased, ‘**=**’ Same as/no change, ‘≠’ No effect, ‘**+**’ and

### Limitations of iPSC-Based Models of Hyperexcitability

As with any model, there are limitations associated with using iPSC to model neurodegenerative disease. Many of these have been reviewed previously, particularly with regard to ageing as a risk factor (Balusu et al. 2023; Giacomelli et al. 2022; Ooi et al. 2020) but there are also limitations specific to modelling hyperexcitability and excitotoxicity. Foremost is that neuronal activity in vivo is affected by surrounding cell types, notably glial cells, which is particularly important when considering non-cell autonomous mechanisms of neurodegeneration. Co-cultures of iPSC-derived neurons, established with either human iPSC-derived glia (Lawson et al. 2023; Lee et al. 2023; Soubannier et al. 2024; Szebényi et al. 2024; Vahsen et al. 2023) or rodent glia (Odawara et al. 2016) have been used to overcome this limitation. However, neuron-glia co-cultures are not yet standard practice for electrophysiology experiments, and while they provide highly useful models, they do not re-produce in vivo cell ratios and anatomical organisation. More recent approaches involve multi-cellular organoid models cultured onto two-dimentional/planar MEAs (Oliva et al. 2024) or three-dimentional MEAs that embed electrodes into organoids or which fold around the organoid (Huang et al. 2022; Soscia et al. 2020; Yang et al. 2024), these models have the advantages of being multi-cellular with organisation similar to in vivo, and through use of assembloids (Miura et al. 2022), can model in vivo anatomical relationships. For ALS, a further and related consideration is that lower motor neurons form synapses with skeletal muscle. iPSC-derived models of the NMJ have been used in combination with patch-clamping and calcium imaging-based approaches to quantify neuronal activity (Andersen et al. 2020; Miura et al. 2022). An alternative approach are microfluidic systems that enable compartmentalisation of axons from the dendrites and neuronal soma. These can be used in co-culture experiments with skeletal muscle cells seeded into the axonal compartment and/or with glial cells seeded into the somatodendritic compartment, but so far as we are aware, such studies have used only calcium imaging to quantify neuronal activitiy in iPSC-derived models of ALS (Stoklund Dittlau et al. 2021, 2023).

A second limitation of using iPSC to model hyperexcitability is the challenge of harmonising neuronal network activity with the molecular composition of the cells within the network. Routine laboratory assays for mRNA expression provide data about the whole population (RT-qPCR, ‘bulk’ RNAseq) that is unable to be mapped back to neuronal activitiy. Single-cell technologies such as single-cell RNAseq can provide detailed information on the cell population, specifically the types and number of various neuronal subtypes within the population, but also do not enable mapping back to functional measures of a neuronal network due to the requirement to dissociate the cells into a single-cell suspension for barcoding. In contrast, immunofluorescence can be performed directly on cells used in patch-clamp or MEA experiments, thereby enabling correlation with neuronal activity; however, this is limited by the relatively few proteins that can be quantified in each experiment, and is inherently reliant on suitability of available antibodies. More specialised techniques such as Patch-seq enable single-cell gene expression profiles from iPSC-derived neurons and thereby correlation of electrophysiological function to the transcriptome (Bardy et al. 2016; van den Hurk et al. 2022). These limitations and varied approaches speak to the need for researchers to consider, in a manner appropriate to the complexity of the model being used, the level of molecular characterisation needed to support conclusions from manipulation of electrical activity or when interrogating models of ALS.

## Conclusion

The use of iPSC-derived neuronal models to explore the mechanisms of hyperexcitability and excitotoxicity will build upon the knowledge gathered through studies using animal models and post-mortem tissue. In this way, iPSC-based studies can bridge the gap between animal models and clinical studies, allowing for a better understanding of pathology and addressing the limitations of translatability between animal models and human disease. Modulation in neuronal activity of iPSC-derived cultures can be measured though electrophysiological techniques like whole cell patch clamping and MEAs. With patch clamping techniques measuring neuronal activity at a single cell resolution and MEAs with the ability to provide population-based measurements over time, a combination of these two techniques can provide a comprehensive picture of the effects of hyperexcitability and excitotoxicity in iPSC-derived neuronal cultures. By inducing and studying excitotoxicity in iPSCs using the approaches outlined in this review, we can hope to better understand the pathological mechanisms underlying ALS and other neurodegenerative diseases which feature excitotoxicity, and identify potential therapeutic targets for these diseases.

## Data Availability

All data supporting the findings of this study are available within the paper.
